# Hinokitiol Protects Cardiomyocyte from Oxidative Damage by Inhibiting GSK3*β*-Mediated Autophagy

**DOI:** 10.1155/2022/2700000

**Published:** 2022-04-04

**Authors:** Hongkai Xiao, Siyu Liang, Qinhong Cai, Jinghu Liu, Liang Jin, Zhengfei Yang, Xiaochao Chen

**Affiliations:** ^1^The Fourth Affiliated Hospital of Guangzhou Medical University, Guangzhou 511300, China; ^2^Zengcheng District People's Hospital of Guangzhou, Guangzhou 511300, China; ^3^Sun Yat-Sen Memorial Hospital, Sun Yat-Sen University, 107 Yan Jiang Xi Road, Guangzhou 510120, China; ^4^Weil Institute of Emergency and Critical Care Research, School of Medicine, Virginia Commonwealth University, Richmond, VA, USA

## Abstract

More and more attention has been paid to the use of traditional phytochemicals. Here, we first verified the therapeutic potential of a natural bioactive compound called Hinokitiol in myocardial ischemia reperfusion injury. Hinokitiol exerts cardioprotective effect through inhibition of GSK-3*β* and subsequent elimination of excessive autophagy, tuning autophagic activity in moderate extent for remedial profit in acute myocardial infarction and myocardial ischemia reperfusion injury. Overall, our study establishes Hinokitiol as a novel available interventional treatment for myocardial ischemia reperfusion injury.

## 1. Introduction

Acute myocardial infarction (AMI) is the leading cause of morbidity and mortality worldwide. As the most acute manifestation of coronary artery disease, AMI leads to an acute ischemia hypoxia and dysfunction of myocardial processes, which finally cause extensive cardiac cell death [1].

During ischemia, the most important strategy is to timely restore the blood flow to the ischemic myocardium and to minimize the myocardial infarction size [2]. Although myocardial reperfusion therapy, such as percutaneous coronary intervention and coronary artery bypass grafting, has been the effective approach for reducing myocardial necrosis, however, reperfusion therapy itself could aggravate oxidative stress, Ca^2+^ overload, inflammation, and subsequent cellular damage of cardiomyocyte [3]. These adverse events collectively called ischemia/reperfusion (I/R) injury [4].

Autophagy is a “self-protective” mechanism for degrading long-lived, damaged, or misfolded proteins, it is the only known way for organelle degradation [5]. Autophagy activated by the nutrient starvation or metabolic stress, cells maintain homeostasis by facilitating the engulfment and degradation of cellular cytoplasm constituents in response to environmental challenging [6]. Autophagy plays a dual role in myocardial ischemia reperfusion injury [7]. On one hand, ischemia threatens cellular survival due to the deprivation of nutrient and oxygen; basic autophagy may serve as a source of intracellular energy and nutrients to provide a temporary cardioprotective effect for threatened myocardium [8]. On the other hand, excessive autophagy may lead to overload self-consumption and caused cell death [9]. During reperfusion, cells dramatically amplify the production of reactive oxygen species (ROS) via ROS-induced ROS release mechanism; the large amount of ROS causes a stressive environment by producing damaged proteins, organelles, and lipid peroxidation, thereby promoting autophagy. It has been reported that catalase is one of the substrate of autophagy degradation [10]. The absence of catalase causes accumulation of H_2_O_2_ and finally leads to autophagy cell death. Thus, targeting autophagy activation may be a positive strategy for preventing reperfusion injury.

Hinokitiol (*β*-thujaplicin) is a natural tropolone-related compound, which is isolated from the wood of cupressaceous plants [11]. Several research has demonstrated that Hinokitiol has various pharmacological activities, such as antibacterial, anticancer, anti-inflammatory, and antioxidant properties [12–15]. Hinokitiol suppresses the growth of various cancer cells by inhibiting cell proliferation, migration, and inducing apoptosis [16]. Hinokitiol was also reported to exhibit anti-inflammatory effect in LPS-stimulated macrophages through inhibiting TNF-*α* production and NF-*κ*B activation [17]. However, the mechanism of Hinokitiol in regulating I/R injury is still unclear.

In this study, we aim to investigate the possible mechanism of Hinokitiol on protecting H_2_O_2_-induced oxidative damaged in cardiomyocytes. Our results demonstrated that Hinokitiol reduced the apoptosis rate via inhibiting autophagy flux. These findings indicated that Hinokitiol may be a promising therapeutic strategy for oxidative damage of cardiomyocyte.

## 2. Materials and Methods

### 2.1. Reagents

Hinokitiol (469521) was purchased from Sigma (St. Louis, MO, USA). Stock solutions of 10 mM Hinokitiol was dissolved in dimethyl sulfoxide (DMSO, Sigma-Aldrich) and stored at -80°C. Cell Counting Kit-8 (CCK-8) was purchased from Dojindo Molecular Technologies Inc. (Kumamoto, Japan). Annexin V-FITC/propidium iodide (PI) apoptosis straining kit was purchased from BD company (American). The mRFP-GFP-LC3 adenovirus construct was obtained from Hanbio Inc. (Shanghai, China). Hochest33258 was purchased from Kaiji Biology (Jiangsu, China). All the antibodies used were as follows: P62 (#88588), Beclin-1 (#3495), caspase-3 (#9664), Bcl-2 (#4223), p-mTOR (#D9C2), and p-GSK3*β* (Ser9) (#9336) were obtained from Cell Signaling Technology (Beverly, USA). P21 (#55643) was purchased from BD PharMingen (New York, USA). GAPDH (60004) was purchased from Proteintech Group, Inc. (USA).

### 2.2. Cell Culture and H_2_O_2_ Treatment

Human cardiomyocyte cell line AC16 was purchased from Cellcook Inc. Cells were thawed from early passage stocks and passage for less than 6 months in a mitogen-free Dulbecco's modified Eagle's medium (DMEM) (Gibco; Thermo Fisher Scientific, Inc.) supplemented with 10% FBS and 1% penicillin/streptomycin (Gibco; Thermo Fisher Scientific, Inc.). Cells were growth in a humidified 5% CO2 incubator at 37°C. To induced autophagy or apoptosis, AC16 cells were incubated with 1 mM (autophagy induction) or 2 mM (apoptosis induction) H_2_O_2_ for 2 h. In addition, the cells were incubated with or without 20 *μ*M Hinokitiol for 30 min before H_2_O_2_ exposure.

### 2.3. Western Blotting

Cells were lysed in SDS buffer containing protease and phosphatase inhibitor cocktails (Thermo Fisher, USA). BCA protein assay kit (Thermo Fisher, USA) was used to measure the protein concentration of the lysate. After normalization, protein extracts were separated by 8%, 10%, 12%, or 15% SDS-PAGE and transferred onto the PVDF membrane (Bio-Rad Laboratories, USA); the membranes were then incubated with primary antibodies at 4°C over night. The next day, blots were incubated with species-specific HRP-conjugated secondary antibodies (Thermo Fisher, USA). Signal quantification was performed by enhanced chemiluminescence (ECL, Pierce).

### 2.4. Cell Viability Detection

Cell viability was evaluated by CCK-8 assay. Briefly, a total of 3000 cells were seeded in 96-well plates for 24 h to adhere. The Hinokitiol group was pretreated with 20 *μ*M Hinokitiol for 30 min; the control group was pretreated with equal volume of DMSO. Consequently, cells were subjected to different concentrations of H_2_O_2_ for 24 h; at the end of this periods, 10 *μ*l CCK-8 reagent was added to each well and incubated for another 4 h. The absorbance (OD value) at 450 nm was measured with a spectrometer (SpectraMax M5 Microplate Reader, Molecular Devices LLC). The IC50 of two groups was determined by the GraphPad Prism 8.0 software.

### 2.5. Flow Cytometry

A total of 1 × 10^6^ cells were seeded in a 6-well plate for 24 h to adhere and treat as indicated. After that, cells were washed with a cold PBS; 5 *μ*l Annexin V-FITC and 5 *μ*l 7AAD were added into the cells and stained for 15 min at room temperature. Apoptotic cells were detected by a Beckman-Coulter Flow Cytometry FC500. Data analyzing was performed by the FlowJo software.

### 2.6. Hochest33258 Staining

AC16 cells (5 × 10^4^) were seeded in 4-well Lak-TeK Chamber slide; after 24 h adhere, cells were treated as indicated. After that, cells were fixed with 4% formaldehyde in phosphate-buffered saline (PBS) for 15 min and then strained with 10 mg/L Hoechst33258 for another 1 h. The number of apoptotic cells was counted in different fields of view using a fluorescence microscopy.

### 2.7. Autophagy Flux Monitoring

Autophagy flux was monitored by mRFP-GFP-LC3 reporter system according to the manufacturer's instructions. Briefly, AC16 cells were transfected with mRFP-GFP-LC3 adenovirus (MOI = 100) for 24 h, and then, cells were treated with Hinokitiol or H_2_O_2_ as indicated. The images were acquired using a fluorescence microscope; both GFP and RFP are detected in autophagosomes, present as yellow puncta, while red puncta (RFP signal only) indicated autolysosomes. Autophagy flux was measured by quantifying the percentage of cells with yellow and red puncta (LC3 positive).

### 2.8. RNA-Sequencing

The small RNA libraries were sequenced on the Illumina sequencing platform by Genedenovo Biotechnology Co., Ltd. (Guangzhou, China).

### 2.9. Microarray Data Sources

The Gene Expression Omnibus (GEO) database (http://www.ncbi.nlm.nih.gov/geo/) was explored, and three independent gene expression datasets were selected for this study (GSE58486, GSE6381, and GSE5406). Gene expression data from 2 different time points (1 day and 5 days postsurgery) in ischia/reperfusion operated animals was obtained from GSE58486. Different stage gene expression of myocardial ischemia-reperfusion was obtained from GSE6381. GSE5406 consisted of human ischemic cardiomyopathy with failing LV myocardium and nonfailing controls.

### 2.10. Bioinformatics Analysis

All statistical analyses were performed using the RStudio (version 1.3.1073) or R software (version 3.6.3). *p* < 0.05 was regarded as statistically significant. Differentially expression analysis was used the “limma” package to identify the differentially expressed mRNA with ∣logFC | >1. Gene set enrichment analysis (GSEA) was performed using R ClusterProfile package and visualized by R gseaplot2 package. Gene set variation analysis (GSVA) was performed to identify predominantly 50 hallmark pathways using the GSVA package (version 1.34.0). GO enrichment was performed using R package GOSemSim (version 2.14.2) [18]. Principal component analysis was perform by R function “prcomp” and visualized by R package (ggplot2 and ggord). The packages of e1071 was applied to calculate SVM-RFE (Support Vector Machine-Recursive Feature Elimination).

## 3. Results

### 3.1. Hinokitiol Protected Cardiomyocytes from H_2_O_2_-Induced Injury

**Figure 1:**
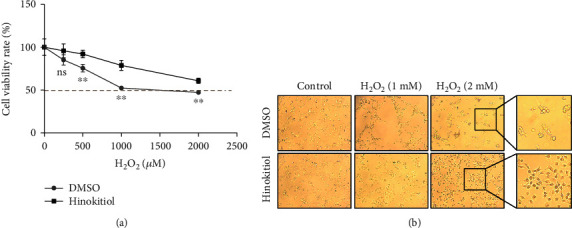
Hinokitiol protected AC16 cells from H2O2-induced damaged. (a) Dose escalation effect of H_2_O_2_ on DMSO or Hinokitiol pretreated AC16 cells, cell viability was measured by CCK-8 assay. ns, *p* > 0.05; ^∗∗^*p* < 0.01. (b) Representative images of AC16 cells under bright field, cells were pretreated with DMSO or Hinokitiol for 30 min and then subjected to H_2_O_2_ for another 4 h; the images were obtained from a fluorescence microscope.

To investigate the antioxidant effect of Hinokitiol, we first performed CCK-8 assay to detect the cell viability on human cardiomyocytes AC16; cells were exposed to different increasing concentrations of H_2_O_2_; after 24 h treatment, H_2_O_2_ markedly inhibited cell proliferation in a dose-dependent manner. However, Hinokitiol pretreatment significant limited the cytotoxic effect of H_2_O_2_ (Figure 1(a)). In addition, H_2_O_2_-treated AC16 cells caused a rounded and shrinking cell shape, which showed an apoptotic-like morphological change, while cells in Hinokitiol treating condition partly prevented such morphological changes (Figure 1(b)). These results indicated that Hinokitiol may protect cardiomyocytes from H_2_O_2_-induced injury.

### 3.2. Hinokitiol Reduced Cell Apoptosis Induced by H_2_O_2_

**Figure 2:**
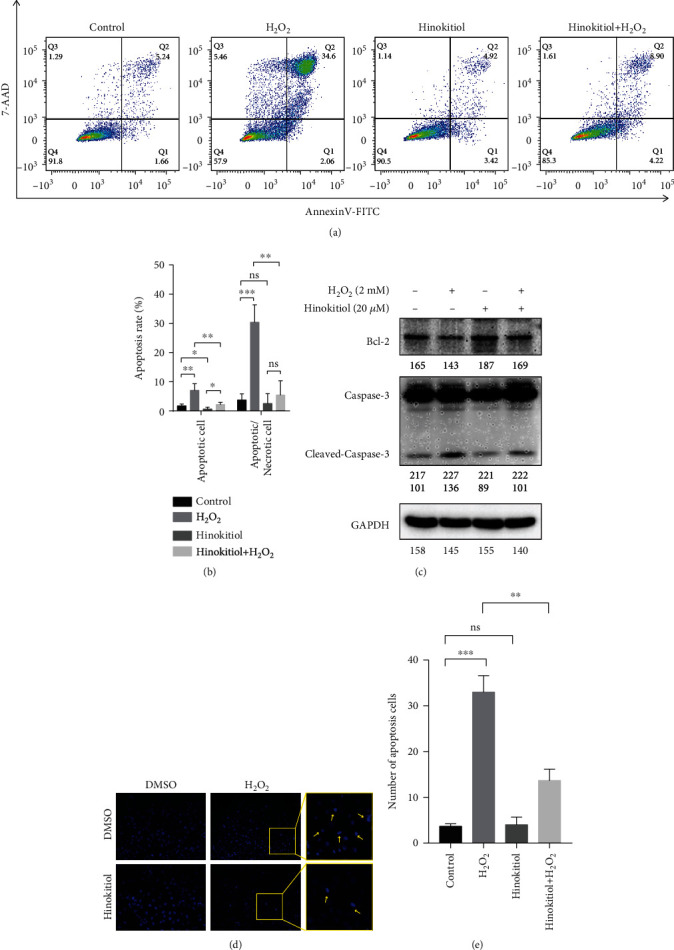
Hinokitiol prevented AC16 cells from H2O2-induced apoptosis. (a) Cells were treated as indicated and straining with Annexin V/7AAD; the apoptosis rate was detected by flow cytometry. (b) Quantification of the percentage of apoptotic rate in (a), ns; *p* > 0.05, ^∗^*p* < 0.05, ^∗∗^*p* < 0.01, ^∗∗∗^*p* < 0.001. (c) Western blotting analysis of AC16 cells treated as indicated. Quantitative analysis of each bands was represented in numerical form under images, respectively. (d) Representative images of AC16 cells after straining with Hochest33258, the yellow arrow indicated apoptotic cells. (e) Quantification of the percentage of apoptotic cell in (d), ns; *p* > 0.05, ^∗∗^*p* < 0.05, ^∗∗∗^*p* < 0.01.

Apoptosis is a typical feature of cardiomyocyte oxidative damage. To further clarify whether Hinokitiol protected cardiomyocytes from H_2_O_2_-induced apoptosis, we used Annexin V/7AAD apoptosis detection kit to stain AC16 cells and analyzed apoptotic ratio by flow cytometry. As Figure 2 shows, the percentage of total apoptosis cell in the H_2_O_2_ group is markedly increased from 6.90% to 36.66% compared to the control group, while Hinokitiol showed limited apoptosis induction on AC16 cells (8.34%). Notely, Hinokitiol pretreatment led to a decrease of apoptosis rate (13.12%) compared to the H_2_O_2_ group (Figures 2(a) and 2(b)). Similarly, western blotting also revealed H_2_O_2_ induced the cleaved and activation of caspase-3, a apoptotic marker, and the reduced expression of antiapoptosis protein Bcl-2, whereas pretreatment with Hinokitiol significantly reversed these changes (Figure 2(c)). Moreover, we stained Hochest33258 to conform the apoptotic features of nuclei in AC16 cells; after H_2_O_2_ treatment, the number of cells with condensed chromatin and apoptotic bodies was significant upregulated, while Hinokitiol treatment protected cells form pyknotic nuclei (Figures 2(d) and 2(e)). These findings demonstrated that Hinokitiol may protect cardiomyocytes from H_2_O_2_-inducd apoptosis.

### 3.3. Hinokitiol Inhibited H_2_O_2_-Mediated Autophagy Flux

**Figure 3:**
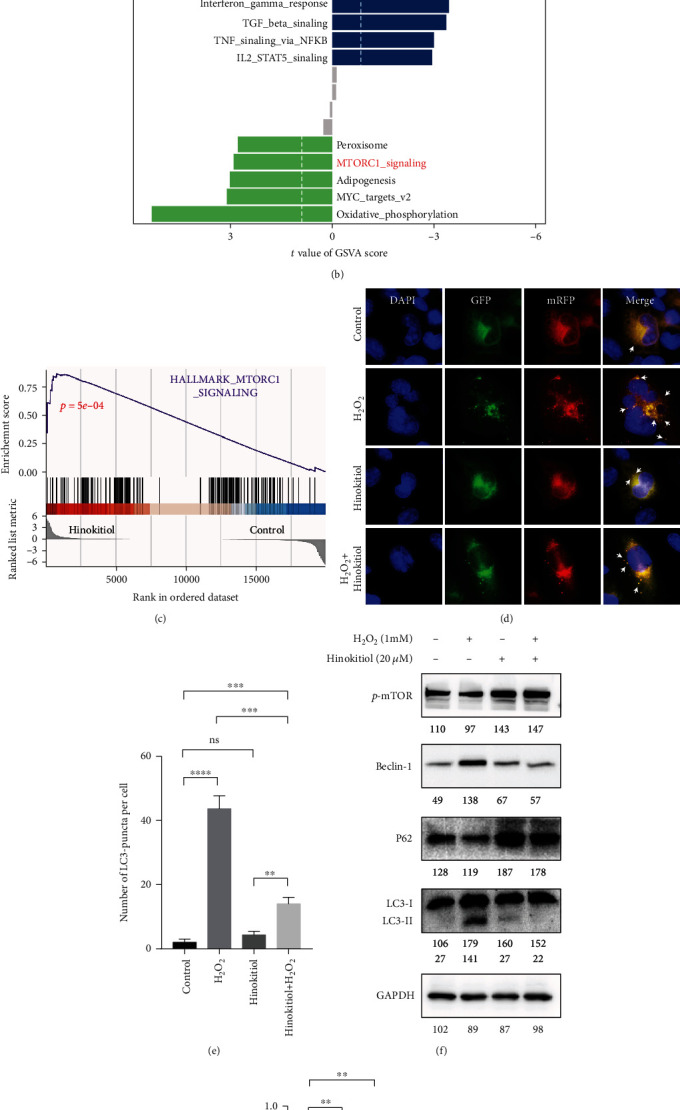
Hinokitiol inhibited autophagy flux induced by H2O2. (a, b) GSVA enrichment of GSE58486 and GSE6381. (c) RNA-sequencing of AC16 cells with different treatment as indicated, the data was analyzed by GSEA enrichment. (d) Detection of autophagic flux by mRFP-GFP-LC3 reporter system in AC16 cells. The microscopy images were merged with DAPI, GFP, and RFP fluorescence of cells. (e) The percentages of yellow or red puncta were calculated and present as histogram, ns; *p* > 0.05; ^∗∗^*p* < 0.01; ^∗∗∗^*p* < 0.001; ^∗∗∗∗^*p* < 0.0001. (f) Protein expression of autophagy-related factor LC3B, Beclin-1, P62, and p-mTOR. Quantitative analysis of each bands was represented in numerical form under images, respectively. (g) The ratio of LC3II/LC3I according to western blotting results. ns, *p* > 0.05; ^∗∗^*p* < 0.01.

In order to clarify the cardioprotective mechanism of Hinokitiol. We obtained two datasets form GEO database and analyzed the data with GSVA enrichment. According to the enrichment results, mTOR pathways were continuously activated during 5 minutes (Figure 3(a)) and 5 days after ischemia (Figure 3(b)), indicating that mTOR may be an important factor in myocardial I/R injury. Subsequently, we confirmed these results in our RNA-sequencing data of Hinokitiol-treated AC16 cells. GSEA enrichment results showed that the mTOR pathway in AC16 cells was activated after Hinokitiol treatment (Figure 3(c)). Since mTOR protein emerged as a negative regulator of autophagy, we next detected the activation of autophagic flux by mRFP-GFP-LC3 reporter system. As presented in Figure 3(d), compared to the control group, autophagy flux was increased after H_2_O_2_ treatment (as indicated by increased of yellow or red puncta), while Hinokitiol pretreatment decreased the activation of autophagy flux induced by H_2_O_2_ (Figure 3(e)). Meanwhile, treatment with H_2_O_2_ significantly increased the LC3B-II/I ratio, which was reversed by Hinokitiol administration (Figures 3(f) and 3(g)). From Figure 3(f), we also found that treatment with H_2_O_2_ significantly increased the level of Beclin-1 and decreased he level of p62 while Hinokitiol administration remarkably lessened these changes.

### 3.4. Hinokitiol Inhibited GSK3*β*-Mediated Autophagy Flux

**Figure 4:**
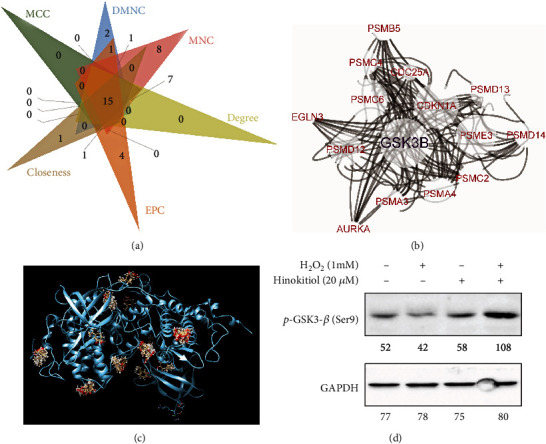
Hinokitiol inhibited GSK3*β*-mediated autophagy flux. (a, b) Venn diagram and PPI network diagram of hubgene. (c) Molecular docking: Hinokitiol potentially binding to the molecular of GSK3*β*. (d) Western blotting analysis: Hinokitiol inhibited GSK3*β* as demonstrated. Quantitative analysis of each bands was represented in numerical form under images, respectively.

Since the two key pathways influenced by Hinokitiol administration in I/R injury are APOPTOSIS and MTORC1_SIGNALING through bioinformatics analysis of GSEA HALLMARK gene sets, we constructed a PPI network of all the genes from these two pathways using STRING v11.0 database (https://string-db.org/). Only the interactions with highest confidence interaction scores defined by the STRING database (≥0.9) were considered. Then, we performed Cytoscape (version 3.7.1) for network visualization and cytohubba app to extract hub genes from the PPI network. Top ten scores of hub genes of these six measures were selected, and the common genes (15 genes) were shown using Venn diagram and PPI network diagram (Figures 4(a) and 4(b)). Furthermore, we performed molecular docking through SwissDock website (http://www.swissdock.ch/), and we found that Hinokitiol potentially binding to the molecular of GSK3*β* (Figure 4(c)). In parallel, Hinokitiol inhibited GSK3*β* through its phosphorylated at Ser9 site as demonstrated through western blot (Figure 4(d)).

### 3.5. Hinokitiol Exerts Antiapoptosis Effect through p21

**Figure 5:**
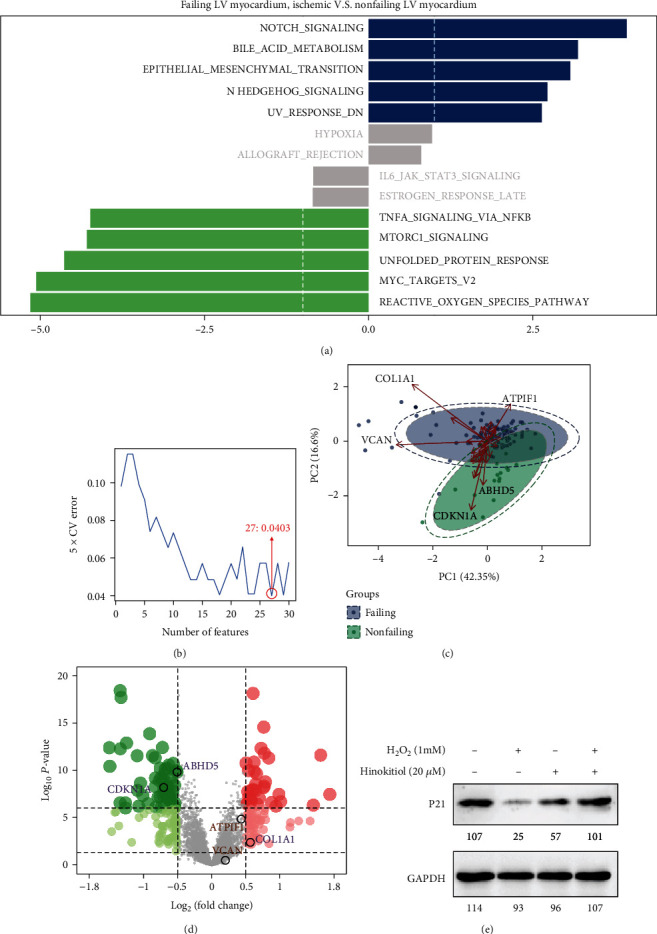
Hinokitiol increased p21 expression by inhibited GSK3*β*. (a) GSVA enrichment of GSE5406. (b) SVM-RFE algorithms in GSE5406 to predict the key genes responsible for heart failure after MI. (c) Partial least square discriminant analysis (PCA) for GSE5406 datasets to identify the vital genes responsible for heart failure after MI. (d) Volcano plot of fold changes of genes with human failing LV myocardium comparison with human nonfailing LV myocardium after MI. *p* values were calculated using the Wald test. (e) Western blotting analysis: Hinokitiol increase p21 expression as demonstrated. Quantitative analysis of each bands was represented in numerical form under images, respectively.

Epidemiologically, approximately one in four patients with myocardial infarction will develop heart failure [19]. Therefore, we conducted pathway enrichment analysis on GEO database (dataset GSE5406) and found that the top five pathways that are significantly upregulated or downregulated in MI patients are accompanied by left ventricular dysfunction (Figure 5(a)). In addition, the packages of e1071 were applied to calculate SVM-RFE [20], and 27 genes were selected for PCA (Figures 5(b) and 5(c)). CDKN1A was identified an important gene which also significantly decrease in patients suffered heart failure (Figure 5(d)). Finally, western blotting was performed to confirm that p21 (encoded by the gene CDKN1A) may had cardiomyocyte protective effect. As a result, p21 decreased in the H_2_O_2_ treatment group, which could be reversed by Hinokitiol intervention (Figure 5(e)).

## 4. Discussion

Accumulated evidence indicates that excessive autophagy mediating by GSK-3*β* activation during reperfusion is detrimental to the myocardium [21, 22]. In this study, we tried to figure out the heart protection properties of Hinokitiol in order to find out a new effective alternative therapeutic interventions.

Previous studies have shown that excess H_2_O_2_ during I/R injury disturbed the balance of intracellular calcium and ROS homeostasis, and its subsequent effects are harmful to the heart during reperfusion [23, 24]. To date, there have been only a few report indicated the protective role of Hinokitiol against H_2_O_2_-induced injury in other cell cultures [25, 26]. Herein, we first found that accumulated H_2_O_2_ was deleterious to AC16 cardiomyocyte with decreased cell viability, while appropriate use of Hinokitiol would lead to the evident alleviation of cardiomyocyte damage (Figure 1). From Figure 1(b), we found that the distribution of AC16 cells was distinctly sparse when treated with 2 mM H_2_O_2_ without Hinokitiol pretreatment. In order to prove the strong protective effect of Hinokitiol on AC16 cells against H_2_O_2_-induced injury, 2 mM H_2_O_2_ was selected for apoptosis detection in Figure 2(c). We also found that the cell morphology was shrinking in the 2 mM H_2_O_2_-treated group without Hinokitiol pretreatment which made it hard to observe in immunofluorescence microscope (the important way to access the autophagy flux). In Figure 3(f), normal cell morphology was needed to assess autophagy flux, and therefore, 1 mM H_2_O_2_ was selected. In addition, p21 and GSK3*β* (shown in Figures 4(d) and 5(e)) were the upstream regulatory factors of autophagy, and the changes of them may occur when apoptosis is not very serious. Taken this factors into account, 1 mM H_2_O_2_ was more suitable for access in Figures 4(d) and 5(e).

Furthermore, we clarified the underlying mechanism of H_2_O_2_-induced myocardial injury and the H_2_O_2_ scavenging function of Hinokitiol. Using Annexin V-FITC/7AAD double-labeled flow cytometry and Hochest33258 straining method, we found that Hinokitiol treatment inhibited H_2_O_2_-mediated cell apoptosis in cardiomyocytes. We also confirmed that cardiomyocyte apoptosis level was remarkably reduced with the intervention of Hinokitiol, as indicated by increased antiapoptotic protein Bcl-2 and decreased cleaved-caspase-3 activity through western blotting (Figure 2). Therefore, Hinokitiol might be a potentially effective therapeutic options for reducing acute myocardial I/R injury.

To determine how Hinokitiol protects cardiomyocytes from oxidant injury at the molecular level, we employed bioinformatic analysis to investigate the GSVA score of hallmark gene sets using microarray datasets GSE6381 and GSE58486 from the GEO database. From early (GSE6381) and late (GSE58486) gene expression profiles, we confirmed that mTORC1-signaling was continuous inhibited during myocardial ischemia-reperfusion (Figures 3(a) and 3(b)).

Consistent with our work, many articles have been confirmed that mammalian target of rapamycin (mTOR), a serine/threonine kinase made up of mTOR complex 1 (mTORC1) and mTOR complex 2 (mTORC2), plays an important role in alleviating myocardial I/R injury and the suppression of mTOR contribute to the excessive autophagy and subsequent I/R injury [27–29]. Transcriptome sequencing was performed to identify the possible mechanism of cardiomyocyte protective effect of Hinokitiol. According to the result of GSEA analysis of transcriptome sequencing, we confirmed that mTORC1 pathways were significantly enriched in the Hinokitiol interference group (Figure 3(c)). Functionally, overexpression of cardiac mTORC1 competently ameliorated myocardial I/R injury and inhibited the adverse myocardial remodeling [30]. Since mTOR is the key suppressor of excessive autophagic flux [31], we further explored the regulatory function of Hinokitiol on autophagy, and we confirmed that excessive autophagy induced by H_2_O_2_ was restrained by Hinokitiol (Figures 3(d)–3(g)). Thus, Hinokitiol administering might be available for attenuating H_2_O_2_-induced cardiac I/R injury.

Antecedent work showed that mTOR activity was enhanced when GSK-3*β* was inhibited, while mTOR activity was inhibited by GSK-3*β* activation [22]. More importantly, inhibition of GSK-3*β* is necessary for mTOR's myocardial protective function to blunt the reperfusion injury [32]. Consistently, through bioinformatics analysis of hub genes, we found that GSK-3*β* is one of the hubgenes in both apoptosis and mTORC1 pathway (Figures 4(a) and 4(b)). Therefore, to figure out if there any direct conjugation between Hinokitiol and GSK-3*β*, we performed molecular docking, and the result indicated that Hinokitiol might binding to GSK-3*β* directly (Figure 4(c)) and subsequently inhibit GSK-3*β*, which confirm by western blotting (Figure 4(d)). It has demonstrated that phosphorylated Ser9 site of GSK-3*β* led to its inhibition and subsequent excessive autophagy elimination [33–35]. These evidences were suggestive that Hinokitiol had the potential to eliminate excessive autophagy during ischemia reperfusion GSK3*β*-mediated autophagy flux and turned autophagy to basal level which was benefit to the myocardial.

P21, encoded by the gene CDKN1A, is a cyclin-dependent kinase (Cdk) inhibitor (CKI) which binds to and inhibits the activity of cyclin-CDK2 or -CDK4 complexes [36]. As an important antiapoptotic protein and regulator of stress-induced premature senescence, cytoplasmic p21 is capable of binding to the procaspase-3 and prevent caspase-3 from activation [37].

Heart failure, a frequent complication of MI, may result from various reasons, such as apoptosis [38, 39]. Downregulated of p21 may responsible for cardiac apoptosis induced by ischemia/reperfusion and subsequent heart failure [40, 41]. It has been reported that inhibition of GSK3*β* was associated with increased expression of p21 [42]. So we speculate that Hinokitiol can increase p21 expression and attenuating H_2_O_2_ induced cardiac I/R injury and we confirmed this by western blotting (Figure 5(e)). These findings provided significant insights into GSK3*β*/p21 signaling due to Hinokitiol intervention which attenuates cardiac apoptosis and exerts cytoprotection simultaneously.

## 5. Conclusions

**Figure 6:**
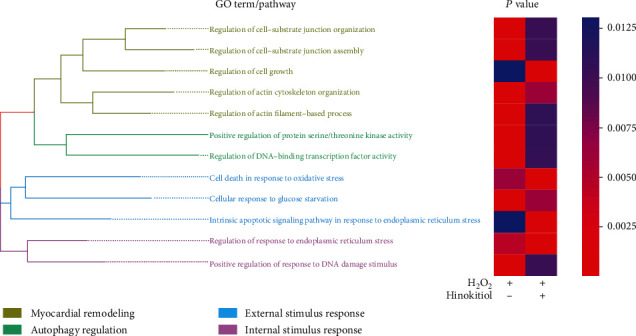
Hinokitiol inhibited GSK3*β*-mediated autophagy flux. Enriched Gene Ontology (GO) of Hinokitiol.

Our study provides the first confirmation of the Hinokitiol as a potential therapeutic option for attenuating I/R injury induced by excessive autophagy. Hinokitiol administering exerts heart protection function via myocardial remodeling, autophagy regulation, external stimulus response, and internal stimulus response-related pathways (Figure 6).

## Data Availability

The raw data supporting the conclusions of this article are available from the corresponding author upon request.

## Abbreviations

AMI: Acute myocardial infarction
ROS: Reactive oxygen species
DMSO: Dimethyl sulfoxide
GFP: Green fluorescent protein
RFP: Red fluorescent protein
CCK-8: Cell counting Kit-8
DAPI: 4′,6-Diamidino-2-phenylindole
GEO: Gene expression omnibus
SVM: Support vector machines
GSEA: Gene set enrichment analysis
GSVA: Gene set variation analysis
PPI: Protein-protein interaction networks
PCA: Principal component analysis.
